# Comparative effectiveness of dexamethasone in treatment of hospitalized COVID-19 patients in the United States during the first year of the pandemic: Findings from the National COVID Cohort Collaborative (N3C) data repository

**DOI:** 10.1371/journal.pone.0294892

**Published:** 2024-03-21

**Authors:** Richard Zhou, Kaitlyn E. Johnson, Justin F. Rousseau, Paul J. Rathouz, on behalf of the N3C Consortium

**Affiliations:** 1 Department of Biomedical Engineering, University of Texas at Austin, Austin, Texas, United States of America; 2 Department of Integrative Biology, The University of Texas at Austin, Austin, Texas, United States of America; 3 The Pandemic Prevention Institute, The Rockefeller Foundation, New York, New York, United States of America; 4 Dell Medical School at the University of Texas at Austin, Austin, Texas, United States of America; Ajou University School of Medicine, REPUBLIC OF KOREA

## Abstract

**Background:**

Dexamethasone was approved for use in hospitalized COVID-19 patients early in the pandemic based on the RECOVERY trial, but evidence is still needed to support its real-world effectiveness in heterogeneous populations of patients with a wide range of comorbidities.

**Methods:**

COVID-19 inpatients represented within the National COVID Cohort Collaborative (N3C) Data Enclave, prior to vaccine availability, were studied. Primary outcome was in-hospital death; secondary outcome was combined in-hospital death and severe outcome defined by use of ECMO or mechanical ventilation. Missing data were imputed with single imputation. Dexamethasone-treated patients were propensity score (PS) matched to non-dexamethasone-treated controls, stratified by remdesivir treatment and based on demographics, baseline laboratory values, comorbidities, and amount of missing data before imputation. Treatment benefit was quantified using logistic regression. Further sensitivity analyses were performed using clinical adjusters in matched groups and in strata defined by quartiles of PS.

**Results:**

Dexamethasone treatment was associated with reduced risk of in-hospital mortality for *n =* 1,263 treated, matched 1:3 to untreated, patients not receiving remdesivir (OR = 0.77, 95% CI: 0.62 to 0.95, p = 0.017), and for *n =* 804 treated, matched 1:1 to untreated, patients receiving remdesivir (OR = 0.74, 95% CI: 0.53 to 1.02, p = 0.054). Treatment showed secondary outcome benefit. In sensitivity analyses, treatment effect generally remained similar with some heterogeneity of benefit across quartiles of PS, possibly reflecting concentration of benefit among the more severely affected.

**Conclusions:**

We add evidence that dexamethasone provides benefit with respect to mortality and severe outcomes in a diverse, national hospitalized sample, prior to vaccine availability.

## 1. Introduction

During the first year of the coronavirus disease 2019 (COVID-19) pandemic, efforts were undertaken to identify effective pharmacological interventions for COVID-19 which were readily available in hospitals for repurposing. Early in the pandemic, the RECOVERY Collaborative Group demonstrated that use of dexamethasone resulted in lower 28-day mortality rates for patients on respiratory support [1]. Dexamethasone is a commonly used glucocorticoid. Early findings, which supported the proposed mechanism of reducing the systemic hyper-inflammatory immune response and thus reducing lung and end-organ damage, made corticosteroids a promising intervention [2–4]. Further evidence, including multiple meta-analyses, has supported the benefits of dexamethasone and other corticosteroids in hospitalized COVID-19 patients for reduction of mortality [5–7]. A small randomized clinical trial in Brazil from September 2020 also supported the benefit of dexamethasone for number of days alive and free from mechanical ventilation for hospitalized patients [8].

Observational studies have covered broader patient populations and have resulted in mixed evidence. In a systematic review and meta-analysis, 73 observational studies showed benefit for corticosteroids. However, an additional 32 studies affording comparison had high patient and between-study heterogeneity and suggested a detrimental effect of corticosteroids on mortality, although both the high heterogeneity and selection bias likely had a critical and confounding role in this result [6]. Furthermore, an observational study of dexamethasone in hospitalized COVID-19 patients not receiving respiratory support showed evidence of potential increased risk of 90-day mortality [9], and these findings appear to have been recapitulated in a more recent meta-analysis of hospitalized patients not receiving oxygen therapy [10]. Comparatively, a cohort study of the effectiveness of combined dexamethasone with remdesivir compared to standard-of-care treatment suggests reduction of mortality even for moderate patients; additionally, progression to mechanical ventilation was reduced [11].

Dexamethasone remains a popular corticosteroid for management of hospitalized COVID-19 patients; by one estimate based on 137,870 hospitalized adult COVID-19 patients in the United States (U.S.) from 1/1/2020 to 2/28/2021, 39.1% received dexamethasone during their hospitalization [12]. In the first year of the pandemic, one study also showed increasing numbers of non-hospitalized patients with COVID-19 being prescribed dexamethasone, despite NIH recommendations [13]. The World Health Organization (WHO) currently recommends the use of corticosteroids for patients with severe or critical COVID-19 only [14].

Many questions remain concerning the use of dexamethasone as a front-line clinical therapy for hospitalized COVID-19 patients, especially in real-world settings with a high degree of heterogeneity [15]. Such real-world evidence is possible at scale through use of large population-based data repositories, curated to the point of common measures across sources, and sufficiently large sample sizes to support meaningful comparisons. In response to this need during the pandemic, the informatics community developed the National COVID Cohort Collaborative (N3C) Data Enclave in the United States (U.S.), enabling secondary analysis of electronic health record (EHR) data related to COVID-19 [16].

In this work, we aim to leverage data from the N3C to estimate the real-world effectiveness of treatment with dexamethasone in hospitalized COVID-19 patients in the U.S. during the first year of the pandemic, with analyses stratified by remdesivir treatment. In addition to the methodological rigor realized through use of a centralized, harmonized, and highly granular EHR data repository, we demonstrate the usage of data imputation to handle missing biomarkers and a matched propensity score approach to handle biomarkers of disease severity with N3C data to generate high quality evidence. Use of the varied comorbidity and laboratory test data available in this resource allows for improved estimation of patient severity and enables an observational study of the efficacy of dexamethasone while minimizing bias. These methods could be applied to similar questions regarding real-world treatment effectiveness of pharmacological interventions within the N3C and beyond, and methods demonstration is an important contribution of our study as well.

## 2. Methods

This is a comparative effectiveness study of dexamethasone treatment in hospitalized COVID-19 patients in the U.S. from the first year of the pandemic (1/1/2020 to 2/23/2021), prior to broad vaccine availability and evolution of major viral variants. It uses a retrospective cohort design based on a curated, national, data repository derived from the electronic health record (EHR). In what follows, we first describe the data and patient inclusion criteria. Then, we describe the imputation of missing data. Lastly, we describe propensity score matching of treated and control groups to control for confounders, and estimation of treatment of effect. The study was determined to be “not human subjects research” by the University of Texas at Austin Institutional Review Board. The overall project is conducted under a Data Use Agreement (DUA) between N3C and the University of Texas (UT) at Austin (PI: PJR) and an N3C-approved Data Use Request (DUR) for this specific study.

### 2.1 Study population and data

#### 2.1.1 Cohort of hospitalized COVID-19 patients from N3C

The N3C is a national, representative, and large repository of EHR data on COVID-19 cases and controls (non-COVID-19 patients) initiated and developed by the National Institutes of Health (NIH) National Center for Advancing Translational Sciences (NCATS) [12, 16]. N3C includes patients seen in diverse clinical settings and geographical regions. We used release version 22 from February 23, 2021, comprising data on over 65 contributing (single- and multi-site) health systems, over three million COVID-19 positive patients, and over nine million patients in total.

The patients used in our analysis from this data version mainly were infected with the primary SARS-CoV-2 variant. The first variant of concern (VOC) was the alpha variant (B.1.1.7), which arose from December 29^th^, 2020, through January 12^th^, 2021, with 76 cases across 12 states as of January 13, 2021. The alpha variant reached 66% of new U.S. infections in April 2021 [17, 18]. Vaccines began distribution in the U.S. during December 2020, primarily for healthcare workers and at-risk individuals. By January 2021, 23 million people in the U.S. were vaccinated. The time period of inclusion in our study slightly overlaps with the vaccine rollout, though our cohort still essentially consists of a non-vaccinated population. As of May 2023, 270 million people (81.3%) in the U.S. have received vaccination [19].

Data for analysis in this report were extracted, manipulated and analyzed in the N3C Data Enclave using Spark SQL (Apache Software Foundation), Python version 3.6 (Python Software Foundation), including PySpark (Apache Software Foundation), and R version 3.5.1 (R Foundation). The N3C’s de-identified dataset was used, which obscures ZIP codes and algorithmically shifts dates of service (while maintaining relative dates of service within each unique cohort member’s trajectory). This project leverages the N3C cohort characterization project, from which our retrospective cohort and patient and severity variables were extracted [20].

#### 2.1.2 Patient and site inclusion criteria and endpoint definition

The time period of inclusion is 1/1/2020 to 2/23/2021, and all patients and their associated encounters (inpatient hospitalization) have an admit and end date (by death or discharge) within this time frame. Patients in the analysis cohort were restricted to adult (over 18 years of age) COVID-19 positive patients with inpatient hospitalizations greater than two days, specifically excluding emergency department visits which did not result in a hospital admission.

Following Bennett et al. [20] for primary clinical outcome (endpoint) in the present investigation, patients were classified by their maximum COVID-19 severity level during their hospitalization. We restricted the cohort to three severity levels: *moderate disease*, consisting of inpatient hospitalizations with no use of extracorporeal membrane oxygenation (ECMO) procedure or invasive mechanical ventilation; *severe disease*, consisting of those receiving either ECMO and/or mechanical ventilation; and disease resulting in either in-hospital *death or discharge to hospice*. By definition, in-hospital death or discharge to hospice can occur during or after the dexamethasone administration period.

The availability of patient laboratory and vital measurements was assessed by provider site. Two sites were removed because there were no laboratory measures available in the data at those sites.

#### 2.1.3 Defining dexamethasone-treated patients and the comparison group

From the patients meeting the foregoing criteria, we identified the subset who had a record of dexamethasone usage during their selected hospitalization. In that subset, we differentiated chronic use of dexamethasone for purposes other than treatment of COVID-19 with use of dexamethasone for treatment of COVID-19 by first removing those with any dexamethasone administered prior to hospitalization. Patients determined as such to have been on long-term dexamethasone therapy for conditions other than COVID-19 were not included in either control or treatment groups. We further restricted the entire analysis cohort to (a) those starting dexamethasone treatment in the first two days of hospitalization (treated group), or (b) those who were not treated with dexamethasone at any time during hospitalization (comparison group). Finally, we removed patients who had records of receiving other corticosteroids (prednisone, methylprednisolone, hydrocortisone) besides dexamethasone from the treatment and comparison groups.

#### 2.1.4 Key variables used in this study

Patient comorbidities, based on conditions in a common comorbidity index (Charlson Comorbidity Index, CCI) [21, 22], demographic variables, and laboratory and vital measurement variables were selected based on their availability and clinical significance for use in the imputation, propensity score (PS) matching, and/or logistic regression models predictive of clinical outcome. For detailed information on the variables used in these models and the selection process, refer to S1A in S1 File.

### 2.2 Statistical analysis

#### 2.2.1 Imputation of missing data

Even while patients, provider sites, and variables were selected to avoid high levels of missingness, the analysis dataset still contained a considerable level of missing data, especially for laboratory values. Excluding participants with missing data can lead to bias in treatment effect estimates and, as such, we replaced missing values with imputed ones. Variables that were never missing by the design of our study include clinical endpoints, treatment with dexamethasone (and also with remdesivir), and provider site. Age was also not ever missing. For comorbidity variables, we assumed that no indication of an existing comorbidity meant the patient was unaffected.

From that point, missingness was handled using the multiple imputation (MI) procedure [23].; however, owing to limitations in the N3C platform, we generated and analyzed only one imputed dataset.

We performed imputation of laboratory measures and race in R using the mice package [24] using the variables listed in S1A in S1 File. Observed continuous laboratory measures were first log-transformed and then Winsorized; i.e., values either greater than the 75th percentile plus three-times the inter-quartile range (IQR), or less than the 25th percentile minus three-times the IQR were shrunk to those two boundaries. The quality of imputation was assessed by comparing observed to imputed distributions within each of the treated and comparison groups. Please see S1B in S1 File for more information on the MI procedure and the model specifications.

#### 2.2.2 Propensity score matching

In observational data comparative effectiveness investigations, the risk of bias due to treatment assignment being confounded with disease severity, and ultimately clinical endpoint, is always a concern. As is commonly done, we employed a propensity score (PS) matching approach to generate a no-corticosteroid comparison group that is closely balanced, in terms of comorbidities and severity at admission, with the dexamethasone treatment group [25]. Separate matching was performed for the treatment and control groups receiving remdesivir and the treatment group and control not receiving remdesivir. Using the MatchIt package, propensity scores were estimated using logistic regression [26]. The 19 variables included in the PS are listed in S1A in S1 File. The log-transform of continuous variables at the imputation stage was reversed before inclusion in the PS model. Control subjects were matched without replacement to dexamethasone-treated units using nearest neighbor matching on the propensity score at a 3:1 (not treated to treated) ratio within the non-remdesivir stratum and at a 1:1 ratio within the remdesivir stratum, owing to a smaller number of patients who received remdesivir without dexamethasone. For the no-remdesivir stratum, no caliper was required to achieve sufficient balance, whereas for the remdesivir stratum, a caliper of 0.65 SD units was required. We confirmed that balance in covariates was attained by computing for each covariate the absolute standardized mean difference between the treatment and control groups; we also compared this difference to that obtained before matching.

#### 2.2.3 Treatment effect estimation

Separately within the pair of matched groups receiving remdesivir and the pair of matched groups not receiving remdesivir, we formally compared the dexamethasone-treated group to the matched control group in two steps. First, we considered analyses stratified by quartile of PS, because the PS is presumably related to disease severity around time of admission, as perceived by the provider(s). For each stratum, we fitted two logistic regression models with dexamethasone (treatment group) indicator as the primary predictor of interest. The first model considered *death or hospice* (versus *moderate or severe disease*) as the outcome. The second model considered the combined endpoint of either *severe disease* (see above, *Patient and site inclusion criteria and endpoint definition*) or *death or hospice referral*, versus *moderate disease*, as the outcome. After having fitted quartile-specific models, in the second step, we fitted an aggregate data model using data from all four quartiles pooled together. For all of these models, we included six adjusters: age, Q-score (for comorbidities; see supplementary material), AST, creatinine, platelet count, and WBC. The four laboratory values were all log (base-2) transformed; in this way, the regression coefficients will be interpretable as log-odds-ratios of the outcome associated with a 2-fold increase in the (log-base-2 transformed) predictor. As discussed, the rationale for including these variables, in addition to the wholesale adjustment provided by the PS matched design, is that they were considered a priori to be strongly predictive of clinical outcome [20]. In addition, the four laboratory values may also have a J- or U-shaped effect. For example, leukocytosis (elevated WBC count) is associated with severe illness/infection, but it is also the case that leukopenia (reduced WBC count) can also be associated with severe illness. As such, we performed sensitivity analyses by re-fitting models including both linear and quadratic versions of the log (base-2) laboratory values.

## 3. Results

### 3.1 Patient characteristics

**Fig 1:**
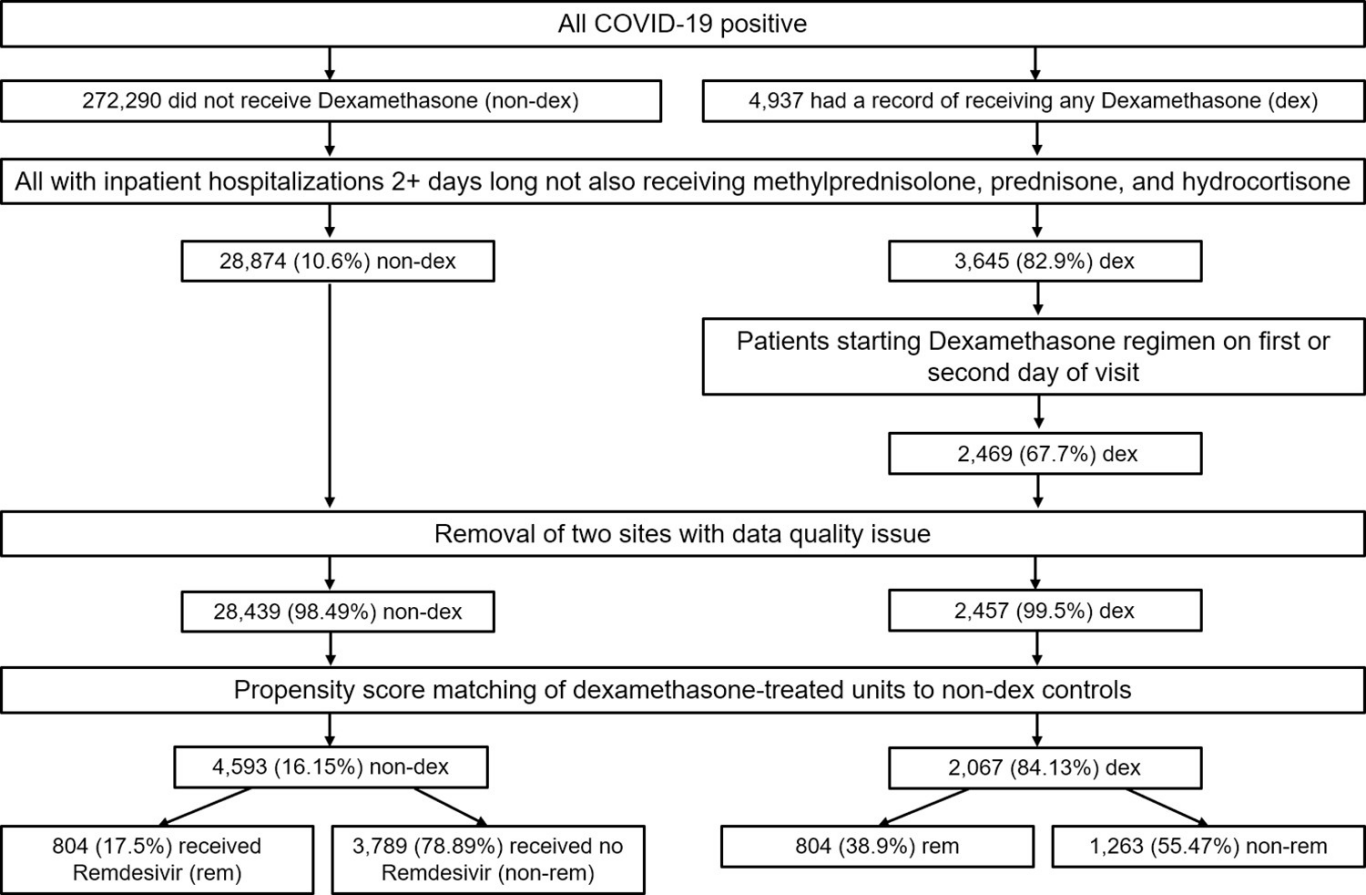
Development of cohort from All N3C patients. Application of inclusion criteria to patients in the N3C and development of matched pairs for comparing the effect of dexamethasone among patients who had and had not received remdesivir, independently groups. Note that within the remdesivir analysis, 390 dexamethasone-treated patients were dropped between the PS-matching and logistic regression stages because there were not enough remdesivir controls with propensity scores similar enough to that of the dexamethasone group.

Fig 1 describes the application of inclusion criteria to develop the study cohort (dexamethasone- and non-dexamethasone-treated patients) from all available patients in the N3C data enclave. Of 4,937 COVID-19 positive patients treated with dexamethasone, 3,645 (82.9%) were inpatient hospitalizations two or more days long where the patient did not also receive prednisone, methylprednisolone, or hydrocortisone. Of these patients, 2,469 received dexamethasone on the first or second day of the visit. After removal of the two sites that had a data quality issue, 2,457 patients remained and were used in imputation and matched to non-dexamethasone treated controls. Of 272,290 non-dexamethasone-treated COVID-19 positive patients, 28,874 (10.6%) were inpatient hospitalizations of length two or more days where the patient did not also receive prednisone, methylprednisolone, or hydrocortisone. After removal of the two sites that had a data quality issue, 28,439 patients remained and were used in imputation and as potential controls during PS matching.

After application of the inclusion criteria, and before imputation and PS matching, there were 2,457 dexamethasone-treated patients, and 28,349 potential non-dexamethasone-treated controls. The dexamethasone-treated group was slightly older (61±16 years) compared to the non-dexamethasone-treated group (56±19 years). Notably, 1,194 patients from the dexamethasone-treated group received remdesivir as part of their treatment, while 1,014 patients from the non-dexamethasone-treated group received remdesivir as part of their treatment. The remaining demographics, laboratory measurements, and comorbidities of these two groups with percentage missing, if applicable, are presented in Table 1.

**Table 1:** Characteristics of dexamethasone-treated patients and potential controls at baseline before imputation and PS matching.

Characteristic	Dexamethasone, N = 2457^1^	Non-Dexamethasone, N = 28,439^1^
Age (years)	61±16	56±19
Sex		
Female	1,121 (46%)	14,588 (51%)
Male	1,336 (54%)	13,851 (49%)
Race		
Asian	58 (3.1%)	792 (3.3%)
Black or African American	687 (37%)	7,544 (32%)
Native Hawaiian or Other Pacific Islander	<20	62 (0.3%)
Other	<20	355 (1.5%)
White	1,106 (59%)	15,132 (63%)
Missing	582 (23.7%)	4,554 (16%)
Remdesivir Received	1,194 (49%)	1,014 (3.6%)
Comorbidities		
Cancer	151 (6.1%)	2,819 (9.9%)
Congestive Heart Failure	293 (12%)	3,856 (14%)
Diabetes Mellitus	690 (28%)	8,274 (29%)
Myocardial Infarction	157 (6.4%)	1,992 (7.0%)
Pulmonary Disease	397 (16%)	5,402 (19%)
Peripheral Vascular Disease	273 (11%)	3,807 (13%)
Q Score	1±2	2±2
Laboratory and Vital Measurements		
BMI (kg/m^2^)	32±9	31±8
Missing	1,087 (44.2%)	6,935 (24.4%)
ALT (IU/L)	42±45	39±56
Missing	836 (34%)	11,483 (40.4%)
AST (IU/L)	54±54	48±57
Missing	653 (26.6%)	9,454 (33.2%)
Albumin (g/dL)	3.59±0.47	3.62±0.59
Missing	657 (26.7%)	9,992 (35.1%)
Creatinine (mg/dL)	1.27±1.09	1.34±1.26
Missing	597 (24.3%)	7,331 (25.8%)
Neutrophils (%)	76±11	71±13
Missing	880 (35.8%)	11,269 (39.6%)
Lymphocytes (%)	16±10	19±11
Missing	682 (27.8%)	10,404 (36.6%)
Platelet Count (x1000/uL)	225±92	228±96
Missing	792 (32.2%)	8,931 (31.4%)
White Blood Cell Count (x1000/uL)	7.8±4.8	8.0±4.8
Missing	603 (24.5%)	7,674 (27%)
Acute Kidney Injury in Hospital^2^	332 (14%)	978 (3.4%)
Smoking Status		
Current or Former	138 (5.6%)	3,040 (11%)
Non smoker	2,319 (94%)	25,399 (89%)

^1^ Statistics presented: mean±SD; n (%).

^2^ AKI was included in imputation

### 3.2 Modeling and outcomes

#### 3.2.1 Imputation and propensity score matching

After imputation, the distribution of imputed and observed values for continuous laboratory values was comparable (S1 Fig).

Propensity score matching of non-dexamethasone-treated controls to dexamethasone-treated patients in both the remdesivir and non-remdesivir groups improved balance. The absolute standardized mean difference between the treated and control group of all continuous covariates and all levels of categorical covariates included in assigning propensity scores was reduced to <0.1 (S2A and S2B Fig). Achieving balance between groups required exclusion of treated units within the dexamethasone and remdesivir treated group which could not be successfully matched to controls. After matching, 2,067 total dexamethasone-treated patients remained. Eight-hundred-four patients were also treated with remdesivir while 1,263 patients were treated with dexamethasone only. Three-hundred-ninety remdesivir and dexamethasone treated patients were dropped between the logistic regression and PS-matching stages because there were not enough controls with propensity scores similar enough to that of the remdesivir and dexamethasone group. In the dexamethasone only group, all units were successfully matched to three controls. The characteristics of the matched groups are summarized in Table 2, with further details in S1 Table.

**Table 2:** Dexamethasone treatment and matched control group summary. For **(A)** the non-remdesivir stratum, characteristics of dexamethasone-treated patients and 3:1 PS matched non-dexamethasone-treated controls, after imputation. For **(B)** the remdesivir stratum, characteristics of dexamethasone-treated patients and 1:1 PS matched non-dexamethasone-treated controls, after imputation. Corresponds with S1 Table. For each laboratory value, the percentage which was imputed is included in S2 Table.

**A) Non-Remdesivir Stratum**		
**Characteristic** ^2^	**Dexamethasone,** N = 1263^1^	**Non-Dexamethasone Matched Controls,** N = 3789^1^
Age (years)	61±16	61±17
Sex		
Female	571 (45%)	1,696 (45%)
Male	692 (55%)	2,093 (55%)
Race		
Asian	52 (4.1%)	171 (4.5%)
Black or African American	501 (40%)	1,496 (39%)
Native Hawaiian or Other Pacific Islander	<20	<20
Other	<20	48 (1.3%)
White	691 (55%)	2,059 (54%)
Comorbidities		
Cancer	88 (7.0%)	260 (6.9%)
Congestive Heart Failure	178 (14%)	523 (14%)
Diabetes Mellitus	379 (30%)	1,143 (30%)
Myocardial Infarction	104 (8.2%)	322 (8.5%)
Pulmonary Disease	234 (19%)	718 (19%)
Peripheral Vascular Disease	157 (12%)	456 (12%)
Q Score	1±2	1±2
Laboratory and Vital Measurements		
BMI (kg/m^2^)	32±9	31±8
ALT (IU/L)	43±57	42±58
AST (IU/L)	56±61	56±73
Albumin (g/dL)	3.57±0.48	3.57±0.57
Creatinine (mg/dL)	1.36±1.23	1.36±1.24
Neutrophils (%)	75±11	76±11
Lymphocytes (%)	16±10	16±10
Platelet Count (x1000/uL)	229±94	230±96
White Blood Cell Count (x1000/uL)	7.9±4.8	8.0±4.0
**B) Remdesivir Stratum**		
**Characteristic** ^2^	**Dexamethasone,** N = 804^1^	**Non-Dexamethasone Matched Controls,** N = 804^1^
Age (years)	62±15	62±16
Sex		
Female	365 (45%)	377 (47%)
Male	439 (55%)	427 (53%)
Race		
Asian	<20	<20
Black or African American	296 (37%)	305 (38%)
Native Hawaiian or Other Pacific Islander	<20	<20
Other	<20	<20
White	478 (59%)	464 (58%)
Comorbidities		
Cancer	50 (6.2%)	56 (7.0%)
Congestive Heart Failure	96 (12%)	102 (13%)
Diabetes Mellitus	242 (30%)	253 (31%)
Myocardial Infarction	45 (5.6%)	55 (6.8%)
Pulmonary Disease	138 (17%)	146 (18%)
Peripheral Vascular Disease	93 (12%)	94 (12%)
Q Score	1±2	1±2
Laboratory and Vital Measurements		
BMI (kg/m^2^)	32±9	33±9
ALT (IU/L)	42±46	41±49
AST (IU/L)	52±52	51±44
Albumin (g/dL)	3.55±0.49	3.53±0.52
Creatinine (mg/dL)	1.18±0.92	1.18±0.86
Neutrophils (%)	75±11	75±11
Lymphocytes (%)	16±10	16±9
Platelet Count (x1000/uL)	221±92	222±83
White Blood Cell Count (x1000/uL)	7.6±4.8	7.6±4.5

^1^ Statistics presented: mean±SD; n (%)

^2^ Cells with patient count less than 20 obscured to protect confidentiality

#### 3.2.2 Effect of dexamethasone

Rates of in-hospital death or referral to hospice were lower in the dexamethasone-treated group compared to the non-dexamethasone-treated matched control group for those receiving remdesivir (OR = 0.74, 95% CI: 0.53 to 1.02), those not receiving remdesivir (OR = 0.77, 95% CI: 0.62 to 0.95), and both remdesivir and non-remdesivir treated groups combined (OR = 0.77, 95% CI: 0.64 to 0.91), although the effect in the smaller remdesivir stratum was only borderline significant.

The use of dexamethasone also was associated, albeit at a weaker level, with a lower incidence of a combined severe outcome or in-hospital death/hospice referral for those receiving remdesivir (OR = 0.83, 95% CI: 0.64 to 1.09), those not receiving remdesivir (OR = 0.84, 95% CI: 0.71 to 1.00), and both remdesivir and non-remdesivir treated strata combined (OR = 0.82, 95% CI: 0.71 to 0.94) (Table 3).

**Table 3:** Outcomes for dexamethasone-treated patients and matched controls. Outcomes and crude odds ratios (95% CI) comparing dexamethasone recipients to PS-matched non-recipients in N3C hospitalized COVID-19 patients. Non-recipients of remdesivir are matched 3:1 (non-dexamethasone to dexamethasone), while remdesivir recipients are matched 1:1.

All Study Patients						
	Dexamethasone Received	Death/Hospice		Severe or Death/Hospice	
**Characteristic** ^3^	**FALSE** N = 4,593^1^	**TRUE** N = 2,067^1^	**OR**	**95% CI**	**OR**	**95% CI**
Maximal Clinical Severity						
Death/Hospice	530 (12%)	188 (9.1%)	0.77	0.64, 0.91	0.82	0.71, 0.94
Severe	312 (6.8%)	133 (6.4%)	ref.			
Moderate^2^	3,751 (82%)	1,746 (84%)			ref.	
ECMO	27 (0.6%)	<20				
Invasive Ventilation	525 (11%)	234 (11%)				
**Non-recipients of remdesivir**						
**Characteristic**	**FALSE** N = 3789^1^	**TRUE** N = 1263^1^	**OR**	**95% CI**	**OR**	**95% CI**
Maximal Clinical Severity						
Death/Hospice	433 (11%)	114 (9.0%)	0.77	0.62, 0.95	0.84	0.71, 1.00
Severe	271 (7.2%)	89 (7.0%)	ref.			
Moderate	3,085 (81%)	1,060 (84%)			ref.	
ECMO	25 (0.7%)	<20				
Invasive Ventilation	437 (12%)	146 (12%)				
**Recipients of remdesivir**						
**Characteristic**	**FALSE** N = 804^1^	**TRUE** N = 804^1^	**OR**	**95% CI**	**OR**	**95% CI**
Maximal Clinical Severity						
Death/Hospice	97 (12%)	74 (9.2%)	0.74	0.53, 1.02	0.83	0.64, 1.09
Severe	41 (5.1%)	44 (5.5%)	ref.			
Moderate	666 (83%)	686 (85%)			ref.	
ECMO	<20	<20				
Invasive Ventilation	88 (11%)	88 (11%)				

^1^ Statistics presented: n (%)

^2^ Moderate is the best possible outcome in this cohort: It represents hospitalization without major complication.

^3^ Cells with patient count less than 20 obscured to protect confidentiality

In sensitivity analyses, the effect of dexamethasone on reduction of in-hospital death/hospice referral (OR = 0.75, 95% CI: 0.59 to 0.95, p = 0.017) and combined severe outcome or in-hospital death/hospice referral (OR = 0.82, 95% CI: 0.68 to 0.98, p = 0.028) in the non-remdesivir stratum remained similar and significant after adjusting for the effect of age, Q-score, AST, creatinine, platelet count, white blood cell count, and PS via stratum indicators for the 4 PS quartiles (Table 4A). Within the strata defined by quartiles of propensity score, the effect of dexamethasone on reduction of both study outcomes in the non-remdesivir stratum after adjustment was stronger in the fourth quartile of propensity scores (presumably patients who presented as most severe) (OR = 0.62, 95% CI: 0.42 to 0.9, p = 0.014 mortality outcome; OR = 0.61, 95% CI: 0.44 to 0.82, p = 0.002, combined severe/mortality outcome) than in the aggregate cohort. In the first through third quartiles, the effect was non-significant and somewhat heterogeneous (S3A Table).

**Table 4:** Effect of dexamethasone in logistic regression models. Prediction of in-hospital death/hospice referral and combined in-hospital death/hospice referral and severe outcome by receipt of dexamethasone with logistic regression models for **(A)** non-remdesivir stratum and **(B)** remdesivir stratum.

**A) Non-Remdesivir Stratum**						
**Aggregate PS Matched Cohort** ^**4**^						
**Characteristic** ^2^ ^,^ ^3^ ^,^ ^4^	**Death/Hospice**			**Severe or Death/Hospice**		
	**OR** ^**1**^	**95% CI** ^1^	**p-value**	**OR** ^**1**^	**95% CI** ^**1**^	**p-value**
Dexamethasone	0.75	0.59, 0.95	0.017	0.82	0.68, 0.98	0.028
Age (years)	1.06	1.05, 1.07	<0.001	1.03	1.02, 1.03	<0.001
Q-Score	1.14	1.09, 1.18	<0.001	1.11	1.07, 1.15	<0.001
AST (IU/L)	1.34	1.22, 1.47	<0.001	1.37	1.27, 1.47	<0.001
Creatinine (mg/dL)	1.27	1.14, 1.42	<0.001	1.21	1.10, 1.32	<0.001
Platelet (x1000/uL)	0.64	0.54, 0.76	<0.001	0.69	0.60, 0.79	<0.001
WBC (x1000/uL)	1.89	1.62, 2.21	<0.001	2.01	1.77, 2.28	<0.001
**B) Remdesivir Stratum**						
**Characteristic** ^**2**^ ^,^ ^**3**^	**Death/Hospice**			**Severe or Death/Hospice**		
	**OR** ^**1**^	**95% CI** ^**1**^	**p-value**	**OR** ^**1**^	**95% CI** ^**1**^	**p-value**
Dexamethasone	0.72	0.51, 1.00	0.054	0.83	0.63, 1.10	0.2
Age (years)	1.06	1.04, 1.07	<0.001	1.02	1.01, 1.03	<0.001
Q-Score	1.06	0.98, 1.14	0.2	1.06	0.99, 1.14	0.070
AST (IU/L)	1.51	1.24, 1.83	<0.001	1.56	1.33, 1.84	<0.001
Creatinine (mg/dL)	1.15	0.89, 1.46	0.3	1.06	0.85, 1.30	0.6
Platelet (x1000/uL)	0.62	0.45, 0.85	0.003	0.63	0.48, 0.82	<0.001
WBC (x1000/uL)	1.45	1.11, 1.88	0.006	1.83	1.47, 2.29	<0.001

^*1*^ OR = Odds Ratio, CI = Confidence Interval. *AST, creatinine, platelet count, and WBC count were log-base-2 transformed.

^*2*^ The model included a categorical variable indicating which PS quartile the patient was in (Q1, Q2, Q3, Q4)

^*3*^ AST, creatinine, platelet count, and WBC count were log-base-2 transformed.

^*4*^ See S3 Table for results within strata defined by quartile of PS

In the remdesivir-treated group, the use of dexamethasone showed a borderline statistically significant benefit in reducing in-hospital death or referral to hospice (OR = 0.72; 95% CI: 0.51 to 1.00, p = 0.054); however, the effect of dexamethasone on the combined severe or in-hospital death/referral to hospice outcome was non-significant (Table 4B). Within strata, in the remdesivir-treated group, effects were largely non-significant (although p = 0.048 in the second quartile) and heterogeneous, likely owing to the small sample sizes (S3b Table).

The expected rationale of the fourth quartile of PS being the most severe was further characterized by assessing the laboratory values by PS. We see trends across laboratory values demonstrating severity differences between quartile of PS for both the remdesivir and non-remdesivir treated groups (S4 Table).

The same benefit of dexamethasone for both the remdesivir and non-remdesivir groups was observed in further sensitivity analyses where the logistic regression models above with linear adjusters were extended to investigate quadratic effects for the laboratory variables included, though the effect of dexamethasone on the combined outcome was again not significant in the remdesivir group (S5 Table).

## 4. Discussion

Our analysis of multi-site EHR data in the first year of the pandemic confirmed existing clinical trial findings that dexamethasone shows in-hospital mortality benefit. We also found that use of dexamethasone results in a reduction in the secondary combination outcome of in-hospital mortality or severe outcome, defined by the use of ECMO or mechanical ventilation. The effect of dexamethasone on reducing the combined severe and in-hospital death/hospice referral outcome was not as strong as the effect on in-hospital death/hospice referral alone. Similar outcomes were observed in the remdesivir-treated and non-remdesivir-treated groups. Although we did not directly assess co-administration of remdesivir with dexamethasone, our analysis suggests that remdesivir did not appear to impact the benefit of dexamethasone in reduction of either mortality or the secondary outcome.

The treatment effects observed in the cohorts were subjected to sensitivity analyses. Treatment effect size was the same when adjusting for both linear and quadratic effects of included covariates; however, we found that the odds ratios for the secondary outcome in the remdesivir groups were non-significant. It is likely that more data are needed in the remdesivir groups to confirm the effect.

Our results are mostly in agreement with existing evidence, but it is important for us to expand on the context of current NIH guidelines and more recent evidence since the first year of the pandemic. Current guidelines strongly recommend dexamethasone as the primary immunomodulator of choice for therapeutic management of hospitalized COVID-19 patients requiring any type of oxygen. Furthermore, a similar study published in April 2023 which also evaluated dexamethasone for inpatients using a national, retrospective, propensity score–weighted cohort study during the first year of the pandemic found significantly reduced odds of mortality or discharge to hospice in those requiring supplemental oxygen or MV and/or ECMO, corroborating our results [27]. Even though our study does not specifically look at benefits in those requiring supplemental oxygen, the findings do not contradict the recommendations. Our cohort is broad, and the study assesses all people who received dexamethasone in this early pre-vaccine period. Our goal was to assess the average effect of treatment among the treated, but we recognize there may be heterogeneity of treatment effects across subpopulations. This is captured in our assessment of treatment effect by quartile of propensity score. In both remdesivir and non-remdesivir groups, there was significant variability of the odds ratios across strata; however, one notable trend was a statistically significant treatment benefit for both study outcomes in the fourth quartile of the non-remdesivir analysis group. As the propensity score is the probability of being assigned treatment given a certain set of covariates, and dexamethasone is more likely to be administered to severe patients, those who have covariates in the PS stratum which suggest a more severe condition at baseline may lead a provider to be more likely to administer dexamethasone. This suggests those in the fourth quartile of PS benefited most from dexamethasone in comparison to other patients receiving dexamethasone, consistent with current practice of use of dexamethasone primarily in more severe patients receiving oxygen support.

Specifically regarding combined remdesivir/dexamethasone therapy, for patients requiring conventional oxygen, there is a moderate recommendation for co-administration of remdesivir with dexamethasone; for patients on high-flow nasal cannula or non-invasive ventilation, there is a weak recommendation for co-administration of remdesivir. No statement is made about remdesivir co-administration for patients already on mechanical ventilation or ECMO [14, 28]. Indeed, evidence remains mixed regarding remdesivir plus dexamethasone treatment. A prospective controlled non-randomized study found a significant reduction in mortality for remdesivir/dexamethasone treatment, compared to dexamethasone alone [29]; however, a retrospective, multicenter cohort study found addition of remdesivir was not associated with shorter hospitalization or in-hospital mortality [30]. Again, our study did not directly assess remdesivir/dexamethasone, or remdesivir, treatment but suggests potentially no reduction of dexamethasone benefit with addition of remdesivir. More specific subgroup analyses directly addressing this issue are likely needed.

The landscape of the pandemic has also changed since the first year of the pandemic, and this includes the emergence of variants of concern (VOCs) and vaccines. We expect efficacy to be generally similar across different variants, because the damaging inflammation (such as cytokine storm, chest/lung inflammation) which is characteristic of the severe immune response triggered by COVID-19 infection is the target of corticosteroid treatment. Furthermore, the emergence of the alpha variant (B.1.1.7) in January 2021 is unlikely to affect our analysis, since while the transmissibility of the alpha variant was found to be higher than the original strain and studies have suggested an increase in mortality in the population due to B.1.1.7 overall [31], a cohort study found no association between SARS-CoV-2 lineage and death for hospitalized patients [32].

It is still unclear how vaccination status is associated with severe/mortality outcomes for hospitalized patients. However, findings have shown that vaccinations are clearly effective for preventing severe COVID-19 infection, that is, preventing a hospitalization in the first place (3.5 to 17.5 times higher hospitalization rates for non-vaccinated) [19].

Regarding limitations, we addressed several statistical issues in the course of our study. First, as a result of treatment guidelines at the time, the number of patients who had received remdesivir in the absence of dexamethasone was not large. The two strata do in principle allow for comparison of dexamethasone treatment effects between those also receiving and those not receiving remdesivir. We did not detect strong evidence of such differences, although our sample size was limited to be able to detect such interaction terms.

Second, regarding concerns about the considerable amount of missing data, we note that other N3C investigators have also struggled with this issue and have nevertheless been able to draw robust and useful conclusions using the N3C platform, and missing data is a fact of life in research based on the electronic health record. A strength of our analysis is that we matched on the number of missing lab values (before imputation) as a predictor of dexamethasone treatment (versus no such treatment). Indeed, before matching, those treated with dexamethasone had far more complete laboratory profiles, most likely reflecting severity of disease. This component of matching may be one of the more powerful variables to achieve balance on severity (which is not directly observed). Also, regarding the use of single imputation, we note that this may result in standard errors being slightly underestimated but should not affect the imputed values themselves.

There are also some other general limitations associated with the use of electronic health record data that have been harmonized across many sites. Key data such as detailed ventilator flow settings, ICU admission, oxygen saturation (SpO_2_), and supplemental oxygen that would have enabled us to answer additional questions about the effects of dexamethasone were not available in the N3C. Specific details about the administration of dexamethasone including delivery route (IV vs oral) and dosage were absent in a portion of patients who received dexamethasone, limiting the potential to study how these factors specifically affect outcomes.

Overall, the large number of sites and patient-level variables available in the N3C were critical in enabling our study and may enable future research in related directions. We also demonstrated the value of the N3C as a resource with the use of methods described in this paper to conduct robust secondary analyses of EHR data producing high quality evidence evaluating the effectiveness of interventions to manage COVID-19. This provides a framework to equip the field to respond quickly to generate evidence to guide management interventions when facing the next emerging, rapidly evolving pandemic when there is not sufficient time to conduct robust prospective clinical trials.

In conclusion, dexamethasone reduced in-hospital mortality and severe outcomes in hospitalized COVID-19 patients; dexamethasone co-administered with remdesivir resulted in similar outcomes. Furthermore, the most severe patients at baseline may benefit most from dexamethasone treatment, as there is likely heterogeneity of treatment effects within the treated group.

## Supporting information

S1 File Supplemental methods. Further information specifying the variables used in the study **(A)** and the multiple imputation methodology **(B)**.
(DOCX)

S1 Table Dexamethasone treatment and matched control group summary. For **(A)** the group consisting of patients *not receiving remdesivir*–characteristics of dexamethasone-treated patients and 3:1 PS matched non-dexamethasone-treated controls, after imputation. For **(B)** the group consisting of patients *receiving remdesivir*–characteristics of dexamethasone-treated patients and 1:1 PS matched non-dexamethasone-treated controls, after imputation. Includes only continuous variables. Shows median and inter-quartile range. Corresponds with Table 2.
(DOCX)

S2 Table Percentage of laboratory values imputed by dexamethasone receipt for remdesivir and non-remdesivir groups. For the group of patients not receiving remdesivir, the percentage of each laboratory value which was imputed, by control group **(A)** and treatment **(B)**. For the group of patients receiving remdesivir, the percentage of each laboratory value which was imputed, by control group **(C)** and treatment **(D)**.
(DOCX)

S3 Table Effect of dexamethasone in logistic regression models, by PS strata. For **(A)** patients *not receiving remdesivir* and **(B)** patients *receiving remdesivir–*prediction of in-hospital death/hospice referral and combined in-hospital death/hospice referral and severe outcome by receipt of dexamethasone with logistic regression models, by strata of PS.
(DOCX)

S4 Table Laboratory values across PS strata. For **(A)** patients *not receiving remdesivir* and **(B)** patients *receiving remdesivir–*median with IQR for each laboratory value, by PS quartile.
(DOCX)

S5 Table Effect of dexamethasone in logistic regression models, with covariate adjustment. Results of extension of models in Table 4 for **(A)** the *non-remdesivir* matched group and **(B)** the *remdesivir* matched group to include quadratic terms for the four log-base-2 transformed laboratory value covariates (creatinine, AST, WBC, and platelet count).
(DOCX)

S1 Fig Imputed versus observed distributions for imputed continuous variables. (TIF)

S2 Fig **A.** Assessment of PS Matching Success for Non-Remdesivir Group. Absolute standardized mean difference before and after 3:1 propensity score matching of non-dexamethasone controls to dexamethasone treated patients. All patients did not receive remdesivir. **B.** Assessment of PS Matching Success for Remdesivir Group. Absolute standardized mean difference before and after 1:1 propensity score matching of non-dexamethasone controls to dexamethasone treated patients. All patients received remdesivir.
(ZIP)
