# Supplementary material for: Comparative effectiveness of dexamethasone in treatment of hospitalized COVID-19 patients in the United States during the first year of the pandemic: Findings from the National COVID Cohort Collaborative (N3C) data repository
Source: PLoS One. 2024 Mar 21;19(3):e0294892. doi: 10.1371/journal.pone.0294892 (PMC10956822; doi:10.1371/journal.pone.0294892)
Supplement: S1 File — Further information specifying the variables used in the study (A) and the multiple imputation methodology (B). (DOCX) [file pone.0294892.s001.docx]

**S1 Supporting Information. Supplemental Methods.** Further information specifying the variables used in the study **(A)** and the multiple imputation methodology **(B)**.

**A) Variables used in this study**

A subset of laboratory and vital sign measurement variables were selected from all those presented in the N3C cohort characterization data tables. These variables were selected based on physician expert (JFR) opinion of clinical significance. Additionally, selected variables were required to have values available for more than 50% of each of the treated and comparison groups at baseline (i.e., within ±2 days of visit start). We used a single measurement for all analyses; in the case of multiple recorded measurement values for a single laboratory test or vital sign for a given patient, we selected for analysis the measurement closest to the visit start. Before propensity score (PS) matching, variables were less likely to be missing for the treated group than for the comparison group. Thus, in PS matching, we included the number (count) of missing laboratory values as an additional matching variable to aid in balancing severity levels between the treated and matched control groups.

The following variables were included as adjusters to account for remaining imbalances in severity at admission in logistic regression models for estimation of treatment effect (see *Treatment effect estimation*, below): age, an updated version of the CCI referred to as Q-score [22], aspartate transaminase (AST), creatinine, platelet count, and white blood cell count (WBC). The rationale for these last four are that AST and creatinine are indicative of end organ damage (hepatic and renal damage, respectively), while platelet count and WBC reflect inflammatory or infectious response.

For the PS matching model and procedure, the six variables above were included, in addition to the following 13 variables: sex, race, receipt of remdesivir during hospitalization, number of selected laboratory measurements missing before imputation, relative percentage of neutrophils, alanine transaminase (ALT), relative percentage of lymphocytes, albumin, and six comorbidities selected based on prior association with poor outcomes in hospitalized COVID-19 patients: congestive heart failure (CHF), diabetes mellitus (DM), peripheral vascular disease (PVD), myocardial infarction (MI), pulmonary disease, and cancer. Once these 19 variables were chosen, imputation was performed to allow for matching to patients based on a full dataset, with missing values imputed.

For imputation, in addition to the 19 variables listed above, we included the following 7 variables to aid in the prediction of missing values: smoking status, acute kidney injury in hospital defined by change in creatinine from baseline (AKI), ECMO received, mechanical ventilation received, length of hospital stay, body mass index (BMI), and maximum severity level during hospitalization. With the exception of smoking status and BMI, these variables were all indicators of in-hospital outcomes; as such, it is not appropriate to include them as PS matching variables, even while they are valid as a basis for imputation of missing values.

**B) Imputation of missing data**

The multiple imputation procedure involves the use of regression on a set of observed values to predict plausible missing values, adding additional noise as would be found in actual, unimputed, data. For each variable with missing values which must be imputed, a separate regression model is fitted. Multiple iterations of imputation are chained, using the predicted values from the past iteration in the regression models of the current iteration, until predicted imputed values begin to converge. Uncertainty is captured by generating multiple imputed datasets; however, owing to limitations in the N3C platform, we generated and analyzed only one imputed dataset.

The following are the settings for the imputation: Variables were imputed using predictive mean matching. Categorical variable race was imputed using polytomous logistic regression. All other variables listed as predictors were complete. Five iterations of the MI algorithm were used to stabilize the conditional "chained equations'' models for prediction of each potentially missing variable. All other settings were defaults in mice.
