# Supplementary material for: Comparative effectiveness of dexamethasone in treatment of hospitalized COVID-19 patients in the United States during the first year of the pandemic: Findings from the National COVID Cohort Collaborative (N3C) data repository
Source: PLoS One. 2024 Mar 21;19(3):e0294892. doi: 10.1371/journal.pone.0294892 (PMC10956822; doi:10.1371/journal.pone.0294892)
Supplement: S1 Table — For (A) the group consisting of patients not receiving remdesivir–characteristics of dexamethasone-treated patients and 3:1 PS matched non-dexamethasone-treated controls, after imputation. For (B) the group consisting of patients receiving remdesivir–characteristics of dexamethasone-treated patients and 1:1 PS matched non-dexamethasone-treated controls, after imputation. Includes only continuous variables. Shows median and inter-quartile range. Corresponds with Table 2. (DOCX) [file pone.0294892.s002.docx]

**S1 Table. Dexamethasone Treatment and Matched Control Group Summary.** For **(A)** the group consisting of patients *not receiving remdesivir* – characteristics of dexamethasone-treated patients and 3:1 PS matched non-dexamethasone-treated controls, after imputation. For **(B)** the group consisting of patients *receiving remdesivir* – characteristics of dexamethasone-treated patients and 1:1 PS matched non-dexamethasone-treated controls, after imputation. Includes only continuous variables. Shows median and inter-quartile range. Corresponds with Table 2.

| **A) Non-Remdesivir Group** | | |
| --- | --- | --- |
| **Characteristic^2^** | **Dexamethasone, N = 1263^1^** | **Non-Dexamethasone Matched Controls, N = 3789^1^** |
| Age | 63 (51, 72) | 63 (50, 74) |
| BMI | 31 (27, 36) | 30 (25, 35) |
| ALT (IU/L) | 30 (18, 47) | 27 (17, 46) |
| AST (IU/L) | 40 (28, 60) | 35 (24, 57) |
| Albumin (g/dL) | 3.60 (3.30, 3.90) | 3.60 (3.20, 4.00) |
| Creatinine (mg/dL) | 1.00 (0.77, 1.40) | 0.98 (0.76, 1.31) |
| Neutrophils (%) | 77 (69, 84) | 78 (69, 84) |
| Lymphocytes (%) | 14 (9, 21) | 14 (9, 21) |
| Platelet Count (x1000/uL) | 212 (166, 276) | 212 (166, 276) |
| White Blood Cell Count (x1000/uL) | 6.9 (5.1, 9.6) | 7.1 (5.3, 9.6) |
| **B) Remdesivir Group** | | |
| **Characteristic^2^** | **Dexamethasone, N = 804^1^** | **Non-Dexamethasone Matched Controls, N = 804^1^** |
| Age | 62 (52, 73) | 62 (51, 74) |
| BMI | 32 (27, 38) | 33 (27, 38) |
| ALT (IU/L) | 42 (20, 50) | 41 (20, 48) |
| AST (IU/L) | 52 (29, 59) | 51 (29, 58) |
| Albumin (g/dL) | 3.55 (3.30, 3.90) | 3.53 (3.20, 3.90) |
| Creatinine (mg/dL) | 1.18 (0.76, 1.24) | 1.18 (0.78, 1.30) |
| Neutrophils (%) | 75 (69, 84) | 75 (68, 84) |
| Lymphocytes (%) | 16 (9, 20) | 16 (9, 21) |
| Platelet Count (x1000/uL) | 221 (161, 263) | 222 (167, 263) |
| White Blood Cell Count (x1000/uL) | 7.6 (4.8, 9.2) | 7.6 (5.1, 8.8) |
| *^1^* Statistics presented: median (IQR) | | |
