# Supplementary material for: Comparative effectiveness of dexamethasone in treatment of hospitalized COVID-19 patients in the United States during the first year of the pandemic: Findings from the National COVID Cohort Collaborative (N3C) data repository
Source: PLoS One. 2024 Mar 21;19(3):e0294892. doi: 10.1371/journal.pone.0294892 (PMC10956822; doi:10.1371/journal.pone.0294892)
Supplement: S2 Table — For the group of patients not receiving remdesivir, the percentage of each laboratory value which was imputed, by control group (A) and treatment (B). For the group of patients receiving remdesivir, the percentage of each laboratory value which was imputed, by control group (C) and treatment (D). (DOCX) [file pone.0294892.s003.docx]

**S2 Table. Percentage of Laboratory Values Imputed by Dexamethasone Receipt (cases and matched controls) for Remdesivir and Non-Remdesivir Groups.** For the group of patients not receiving remdesivir, the percentage of each laboratory value which was imputed, by control group **(A)** and treatment **(B)**. For the group of patients receiving remdesivir, the percentage of each laboratory value which was imputed, by control group **(C)** and treatment **(D)**.

| **Characteristic** | **Missing Count** | **%** |
| --- | --- | --- |
| **A) Non-Remdesivir, Controls (N = 3789)** | | |
| AST (IU/L) | 721 | 13.2781 |
| ALT (IU/L) | 803 | 14.7882 |
| Creatinine (mg/dL) | 524 | 9.6501 |
| Neutrophils (%) | 1046 | 19.2634 |
| Platelet Count (x1000/uL) | 769 | 14.1621 |
| White Blood Cell Count (x1000/uL) | 614 | 11.3076 |
| Lymphocytes (%) | 953 | 17.5506 |
| **B) Non-Remdesivir, Dexamethasone (N = 1263)** | | |
| AST (IU/L) | 238 | 13.6782 |
| ALT (IU/L) | 241 | 13.8506 |
| Creatinine (mg/dL) | 186 | 10.6897 |
| Neutrophils (%) | 395 | 22.7011 |
| Platelet Count (x1000/uL) | 258 | 14.8276 |
| White Blood Cell Count (x1000/uL) | 187 | 10.7471 |
| Lymphocytes (%) | 235 | 13.5057 |
| **C) Remdesivir, Controls (N = 804)** | | |
| AST (IU/L) | 111 | 10.2304 |
| ALT (IU/L) | 109 | 10.0461 |
| Creatinine (mg/dL) | 102 | 9.4009 |
| Neutrophils (%) | 326 | 30.0461 |
| Platelet Count (x1000/uL) | 198 | 18.2488 |
| White Blood Cell Count (x1000/uL) | 108 | 9.9539 |
| Lymphocytes (%) | 131 | 12.0737 |
| **D) Remdesivir, Dexamethasone (N = 804)** | | |
| AST (IU/L) | 140 | 12.0794 |
| ALT (IU/L) | 143 | 12.3382 |
| Creatinine (mg/dL) | 138 | 11.9068 |
| Neutrophils (%) | 193 | 16.6523 |
| Platelet Count (x1000/uL) | 239 | 20.6212 |
| White Blood Cell Count (x1000/uL) | 141 | 12.1657 |
| Lymphocytes (%) | 165 | 14.2364 |
