# Supplementary material for: Comparative effectiveness of dexamethasone in treatment of hospitalized COVID-19 patients in the United States during the first year of the pandemic: Findings from the National COVID Cohort Collaborative (N3C) data repository
Source: PLoS One. 2024 Mar 21;19(3):e0294892. doi: 10.1371/journal.pone.0294892 (PMC10956822; doi:10.1371/journal.pone.0294892)
Supplement: S3 Table — For (A) patients not receiving remdesivir and (B) patients receiving remdesivir–prediction of in-hospital death/hospice referral and combined in-hospital death/hospice referral and severe outcome by receipt of dexamethasone with logistic regression models, by strata of PS. (DOCX) [file pone.0294892.s004.docx]

**S3 Table. Effect of Dexamethasone in Logistic Regression Models, by PS Strata.** For **(A)** patients *not receiving remdesivir* and **(B)** patients *receiving remdesivir* – prediction of in-hospital death/hospice referral and combined in-hospital death/hospice referral and severe outcome by receipt of dexamethasone with logistic regression models, by strata of PS.

| **A) Non-Remdesivir Group** | | | | | | | | | | | | |  |
| --- | --- | --- | --- | --- | --- | --- | --- | --- | --- | --- | --- | --- | --- |
| **Characteristic*** | | **Death/Hospice** | | | | | **Severe or Death/Hospice** | | | | | |  |
|  | | **OR^1^** | | **95% CI^1^** | **p-value** | | **OR^1^** | | **95% CI^1^** | | **p-value** | |  |
| **1st Quartile PS** | | | | | | | | | | | | |  |
| Dexamethasone | | 0.59 | | 0.32, 1.06 | 0.089 | | 0.86 | | 0.54, 1.33 | | 0.5 | |  |
| Age | | 1.06 | | 1.04, 1.08 | <0.001 | | 1.04 | | 1.03, 1.05 | | <0.001 | |  |
| Q-Score | | 1.14 | | 1.06, 1.22 | <0.001 | | 1.13 | | 1.06, 1.20 | | <0.001 | |  |
| AST | | 0.83 | | 0.61, 1.11 | 0.2 | | 1.16 | | 0.94, 1.44 | | 0.2 | |  |
| Creatinine | | 1.25 | | 0.96, 1.60 | 0.093 | | 1.09 | | 0.87, 1.35 | | 0.4 | |  |
| Platelets | | 0.47 | | 0.32, 0.69 | <0.001 | | 0.60 | | 0.44, 0.83 | | 0.002 | |  |
| WBC | | 2.16 | | 1.57, 2.99 | <0.001 | | 2.05 | | 1.57, 2.69 | | <0.001 | |  |
| **2nd Quartile PS** | | | | | | | | | | | | |  |
| Dexamethasone | | 0.77 | | 0.45, 1.27 | 0.3 | | 0.77 | | 0.51, 1.13 | | 0.2 | |  |
| Age | | 1.06 | | 1.04, 1.08 | <0.001 | | 1.03 | | 1.02, 1.04 | | <0.001 | |  |
| Q-Score | | 1.12 | | 1.03, 1.21 | 0.008 | | 1.12 | | 1.05, 1.20 | | <0.001 | |  |
| AST | | 1.47 | | 1.17, 1.85 | 0.001 | | 1.46 | | 1.22, 1.74 | | <0.001 | |  |
| Creatinine | | 1.28 | | 1.00, 1.61 | 0.044 | | 1.18 | | 0.98, 1.42 | | 0.080 | |  |
| Platelets | | 0.69 | | 0.46, 1.04 | 0.074 | | 0.80 | | 0.59, 1.10 | | 0.2 | |  |
| WBC | | 1.99 | | 1.42, 2.80 | <0.001 | | 2.11 | | 1.61, 2.76 | | <0.001 | |  |
| **3rd Quartile PS** | | | | | | | | | | | | |  |
| Dexamethasone | | 1.24 | | 0.78, 1.96 | 0.4 | | 1.20 | | 0.84, 1.69 | | 0.3 | |  |
| Age | | 1.06 | | 1.04, 1.08 | <0.001 | | 1.02 | | 1.01, 1.04 | | <0.001 | |  |
| Q-Score | | 1.13 | | 1.02, 1.24 | 0.017 | | 1.07 | | 0.98, 1.16 | | 0.11 | |  |
| AST | | 1.36 | | 1.10, 1.68 | 0.004 | | 1.39 | | 1.18, 1.63 | | <0.001 | |  |
| Creatinine | | 1.55 | | 1.24, 1.94 | <0.001 | | 1.36 | | 1.13, 1.63 | | 0.001 | |  |
| Platelets | | 0.77 | | 0.53, 1.11 | 0.2 | | 0.66 | | 0.50, 0.88 | | 0.005 | |  |
| WBC | | 2.31 | | 1.64, 3.28 | <0.001 | | 2.55 | | 1.95, 3.34 | | <0.001 | |  |
| **4th Quartile PS** | |  | |  |  | |  | |  | |  | |  |
| Dexamethasone | | 0.62 | | 0.42, 0.90 | 0.014 | | 0.61 | | 0.44, 0.82 | | 0.002 | |  |
| Age | | 1.06 | | 1.04, 1.07 | <0.001 | | 1.02 | | 1.01, 1.03 | | <0.001 | |  |
| Q-Score | | 1.15 | | 1.05, 1.25 | 0.002 | | 1.07 | | 0.99, 1.16 | | 0.087 | |  |
| AST | | 1.44 | | 1.27, 1.64 | <0.001 | | 1.36 | | 1.22, 1.51 | | <0.001 | |  |
| Creatinine | | 1.10 | | 0.91, 1.33 | 0.3 | | 1.20 | | 1.02, 1.42 | | 0.029 | |  |
| Platelet | | 0.65 | | 0.50, 0.86 | 0.002 | | 0.72 | | 0.57, 0.91 | | 0.006 | |  |
| WBC | | 1.47 | | 1.12, 1.94 | 0.006 | | 1.59 | | 1.26, 2.01 | | <0.001 | |  |
| **B)** **Remdesivir Group** | | | | | | | | | | | | |  |
| **Characteristic*** | | **Death/Hospice** | | | | | | | **Severe or Death/Hospice** | | | | |
|  | | **OR^1^** | | **95% CI^1^** | | | **p-value** | | **OR^1^** | | **95% CI^1^** | | **p-value** |
| **1st Quartile PS** | | | | | | | | | | | | | |
| Dexamethasone | | 0.88 | | 0.47, 1.65 | | | 0.7 | | 1.02 | | 0.62, 1.67 | | >0.9 |
| Age | | 1.05 | | 1.03, 1.08 | | | <0.001 | | 1.02 | | 1.00, 1.03 | | 0.062 |
| Q-Score | | 0.98 | | 0.85, 1.11 | | | 0.7 | | 1.02 | | 0.91, 1.14 | | 0.7 |
| AST | | 1.29 | | 0.90, 1.87 | | | 0.2 | | 1.26 | | 0.95, 1.68 | | 0.11 |
| Creatinine | | 1.38 | | 0.93, 2.03 | | | 0.10 | | 1.12 | | 0.81, 1.55 | | 0.5 |
| Platelet | | 0.50 | | 0.28, 0.88 | | | 0.017 | | 0.75 | | 0.47, 1.18 | | 0.2 |
| WBC | | 1.22 | | 0.79, 1.85 | | | 0.4 | | 1.54 | | 1.09, 2.19 | | 0.013 |
| **2nd Quartile PS** | | | | | | | | | | | | | |
| Dexamethasone | | 0.35 | | 0.16, 0.73 | | | 0.007 | | 0.54 | | 0.29, 0.98 | | 0.048 |
| Age | | 1.06 | | 1.03, 1.10 | | | <0.001 | | 1.02 | | 1.00, 1.05 | | 0.042 |
| Q-Score | | 1.08 | | 0.91, 1.26 | | | 0.3 | | 1.03 | | 0.88, 1.18 | | 0.7 |
| AST | | 1.41 | | 0.94, 2.11 | | | 0.10 | | 1.48 | | 1.05, 2.09 | | 0.025 |
| Creatinine | | 1.04 | | 0.58, 1.77 | | | >0.9 | | 1.18 | | 0.73, 1.85 | | 0.5 |
| Platelet | | 0.73 | | 0.36, 1.48 | | | 0.4 | | 0.50 | | 0.27, 0.92 | | 0.028 |
| WBC | | 1.13 | | 0.62, 2.07 | | | 0.7 | | 1.64 | | 0.98, 2.79 | | 0.064 |
| **3rd Quartile PS** | | | | | | | | | | | | | |
| Dexamethasone | | 0.76 | | 0.37, 1.55 | | | 0.5 | | 0.63 | | 0.34, 1.16 | | 0.14 |
| Age | | 1.08 | | 1.05, 1.12 | | | <0.001 | | 1.05 | | 1.03, 1.08 | | <0.001 |
| Q-Score | | 1.12 | | 0.96, 1.29 | | | 0.11 | | 1.17 | | 1.02, 1.33 | | 0.021 |
| AST | | 2.04 | | 1.32, 3.20 | | | 0.002 | | 2.24 | | 1.55, 3.28 | | <0.001 |
| Creatinine | | 0.55 | | 0.28, 1.02 | | | 0.073 | | 0.74 | | 0.44, 1.20 | | 0.2 |
| Platelet | | 0.72 | | 0.37, 1.42 | | | 0.3 | | 0.57 | | 0.32, 1.00 | | 0.051 |
| WBC | | 2.03 | | 1.10, 3.79 | | | 0.024 | | 2.48 | | 1.45, 4.34 | | 0.001 |
| **4th Quartile PS** | | | | | | | | | | | | | |
| Dexamethasone | | 0.95 | | 0.46, 1.96 | | | 0.9 | | 1.21 | | 0.66, 2.24 | | 0.5 |
| Age | | 1.04 | | 1.02, 1.08 | | | 0.004 | | 1.02 | | 0.99, 1.04 | | 0.2 |
| Q-Score | | 1.10 | | 0.88, 1.34 | | | 0.4 | | 1.12 | | 0.92, 1.34 | | 0.2 |
| AST | | 1.77 | | 1.21, 2.59 | | | 0.003 | | 1.89 | | 1.37, 2.66 | | <0.001 |
| Creatinine | | 1.66 | | 0.99, 2.76 | | | 0.051 | | 1.21 | | 0.75, 1.93 | | 0.4 |
| Platelet | | 0.47 | | 0.23, 0.98 | | | 0.043 | | 0.54 | | 0.29, 1.00 | | 0.052 |
| WBC | | 2.01 | | 1.11, 3.67 | | | 0.022 | | 2.65 | | 1.61, 4.45 | | <0.001 |
| *^1^* OR = Odds Ratio, CI = Confidence Interval. *AST, creatinine, platelet count, and WBC count were log-base-2 transformed. | | | | | | | | | | | | | |
