# Supplementary material for: Comparative effectiveness of dexamethasone in treatment of hospitalized COVID-19 patients in the United States during the first year of the pandemic: Findings from the National COVID Cohort Collaborative (N3C) data repository
Source: PLoS One. 2024 Mar 21;19(3):e0294892. doi: 10.1371/journal.pone.0294892 (PMC10956822; doi:10.1371/journal.pone.0294892)
Supplement: S4 Table — For (A) patients not receiving remdesivir and (B) patients receiving remdesivir–median with IQR for each laboratory value, by PS quartile. (DOCX) [file pone.0294892.s005.docx]

| Lab | Q1 | Q2 | Q3 | Q4 |
| --- | --- | --- | --- | --- |
| (A) Non-Remdesivir | | | | |
| val_Albumin_g_per_dL | 3.8 (3.4, 4.1) | 3.7 (3.3, 4) | 3.6 (3.2, 3.9) | 3.4 (3.1, 3.7) |
| val_ALT_SGPT_IU_per_L | 26 (16, 43) | 26 (17, 43) | 28 (17, 45.75) | 30 (18, 54) |
| val_AST_SGOT_IU_per_L | 30 (22, 45) | 33 (23, 50) | 37 (26.5, 59) | 47 (31, 79.5) |
| val_Creatinine_mg_per_dL | 0.9 (0.7, 1.16) | 0.93 (0.74, 1.3) | 1 (0.8, 1.39) | 1.1 (0.83, 1.53) |
| val_Lymphocytes_relative_pct | 19.9 (13, 28.4) | 16 (10, 22.05) | 12.2 (8.2, 17.4) | 10 (6.7, 14) |
| val_Neutrophils_relative_pct | 68 (59, 76.05) | 74 (67.52, 81) | 79.6 (73.2, 85) | 83.8 (78.6, 88) |
| val_Platelet_count_x10E3_per_uL | 218 (168, 276.5) | 209 (164, 272) | 210.67 (167, 278) | 211 (164, 275.75) |
| val_WBCC_x10E3_per_uL | 6.84 (5, 9.8) | 6.7 (5.12, 9.3) | 7.27 (5.5, 9.8) | 7.3 (5.5, 9.6) |
| (B) Remdesivir Group | | | | |
| val_Albumin_g_per_dL | 3.3 (2.95, 3.6) | 3.5 (3.2, 3.8) | 3.7 (3.4, 4) | 3.8 (3.4, 4.1) |
| val_ALT_SGPT_IU_per_L | 27.5 (18.5, 42.75) | 28 (20, 46.37) | 32.67 (21, 52) | 33 (21, 52) |
| val_AST_SGOT_IU_per_L | 38 (28, 55.75) | 40 (29, 61) | 40 (30, 61.75) | 42.5 (31, 58) |
| val_Creatinine_mg_per_dL | 1.06 (0.8, 1.52) | 0.95 (0.75, 1.21) | 0.94 (0.77, 1.2) | 0.89 (0.72, 1.17) |
| val_Lymphocytes_relative_pct | 17 (10, 25.7) | 14.8 (9.05, 20.7) | 13.42 (8.72, 18.37) | 13.1 (8.42, 18) |
| val_Neutrophils_relative_pct | 72.6 (62.05, 81.07) | 75.7 (68, 83.28) | 77.9 (71.7, 84) | 79.5 (72.3, 84.45) |
| val_Platelet_count_x10E3_per_uL | 216 (163.1, 275) | 214 (169, 265) | 201.5 (157.12, 259) | 204.75 (166, 257.75) |
| val_WBCC_x10E3_per_uL | 6.9 (5.02, 9.3) | 6.86 (4.9, 8.8) | 6.6 (4.81, 8.79) | 6.76 (5.1, 9.14) |

**S4 Table. Laboratory Values Across PS Strata.** For **(A)** patients *not receiving remdesivir* and **(B)** patients *receiving remdesivir –* median with IQR for each laboratory value, by PS quartile.
