# Supplementary material for: Comparative effectiveness of dexamethasone in treatment of hospitalized COVID-19 patients in the United States during the first year of the pandemic: Findings from the National COVID Cohort Collaborative (N3C) data repository
Source: PLoS One. 2024 Mar 21;19(3):e0294892. doi: 10.1371/journal.pone.0294892 (PMC10956822; doi:10.1371/journal.pone.0294892)
Supplement: S5 Table — Results of extension of models in Table 4 for (A) the non-remdesivir matched group and (B) the remdesivir matched group to include quadratic terms for the four log-base-2 transformed laboratory value covariates (creatinine, AST, WBC, and platelet count). (DOCX) [file pone.0294892.s006.docx]

**S5 Table. Effect of Dexamethasone in Logistic Regression Models, with Covariate Adjustment.** Results of extension of models in table 4 for **(A)** the *non-remdesivir* matched group and **(B)** the *remdesivir* matched group to include quadratic terms for the four log-base-2 transformed laboratory value covariates (creatinine, AST, WBC, and platelet count).

| **A) Non-Remdesivir Group** | | | | | | |
| --- | --- | --- | --- | --- | --- | --- |
| **Characteristic** | **Death/Hospice** | | | **Severe or Death/Hospice** | | |
|  | **OR^1^** | **95% CI^1^** | **p-value** | **OR^1^** | **95% CI^1^** | **p-value** |
| **Aggregate PS Matched Cohort** | | | | | | |
| Dexamethasone | 0.75 | 0.59, 0.94 | 0.016 | 0.79 | 0.66, 0.95 | 0.013 |
| **1st Quartile PS** | | | | | | |
| Dexamethasone | 0.58 | 0.31, 1.05 | 0.084 | 0.86 | 0.54, 1.34 | 0.5 |
| **2nd Quartile PS** | | | | | | |
| Dexamethasone | 0.75 | 0.44, 1.25 | 0.3 | 0.76 | 0.51, 1.12 | 0.2 |
| **3rd Quartile PS** | | | | | | |
| Dexamethasone | 1.24 | 0.77, 1.97 | 0.4 | 1.20 | 0.84, 1.70 | 0.3 |
| **4th Quartile PS** | | | | | | |
| Dexamethasone | 0.59 | 0.40, 0.86 | 0.008 | 0.57 | 0.41, 0.77 | <0.001 |
| **B) Remdesivir Group** | | | | | | |
| **Characteristic** | **Death/Hospice** | | | **Severe or Death/Hospice** | | |
|  | **OR^1^** | **95% CI^1^** | **p-value** | **OR^1^** | **95% CI^1^** | **p-value** |
| **Aggregate PS Matched Cohort** | | | | | | |
| Dexamethasone | 0.69 | 0.49, 0.97 | 0.034 | 0.79 | 0.60, 1.05 | 0.11 |
| **1st Quartile PS** | | | | | | |
| Dexamethasone | 0.76 | 0.39, 1.46 | 0.4 | 0.88 | 0.52, 1.47 | 0.6 |
| **2nd Quartile PS** | | | | | | |
| Dexamethasone | 0.35 | 0.15, 0.73 | 0.007 | 0.52 | 0.27, 0.95 | 0.036 |
| **3rd Quartile PS** | | | | | | |
| Dexamethasone | 0.73 | 0.35, 1.50 | 0.4 | 0.64 | 0.34, 1.18 | 0.2 |
| **4th Quartile PS** | | | | | | |
| Dexamethasone | 0.98 | 0.47, 2.05 | >0.9 | 1.24 | 0.67, 2.30 | 0.5 |
| *^1^* OR = Odds Ratio, CI = Confidence Interval | | | | | | |
