# Supplementary figures and images for: Comparative effectiveness of dexamethasone in treatment of hospitalized COVID-19 patients in the United States during the first year of the pandemic: Findings from the National COVID Cohort Collaborative (N3C) data repository
Source: PLoS One. 2024 Mar 21;19(3):e0294892. doi: 10.1371/journal.pone.0294892 (PMC10956822; doi:10.1371/journal.pone.0294892)

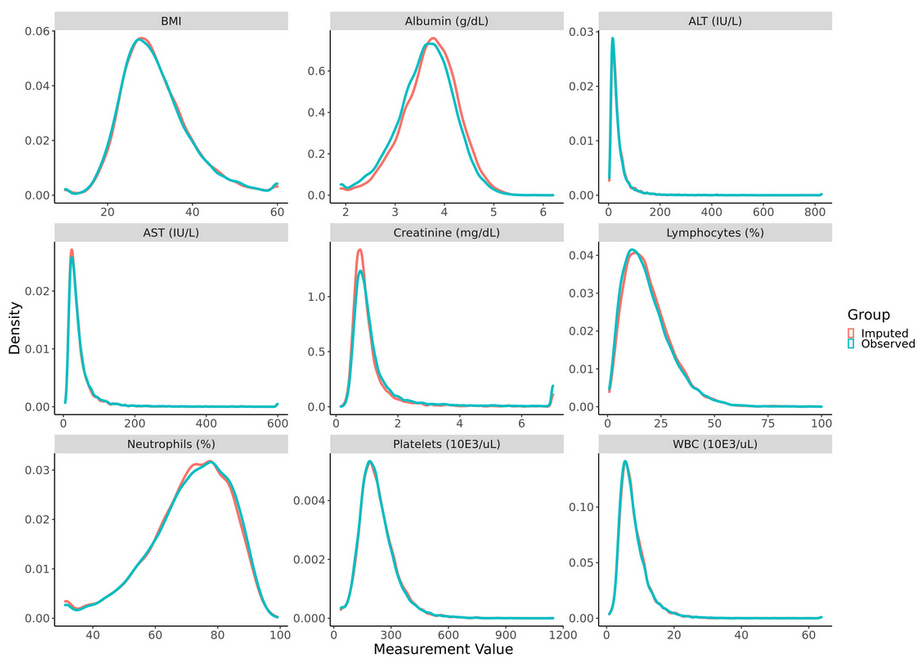

Supplement: S1 Fig — (TIF) [file pone.0294892.s007.tif]

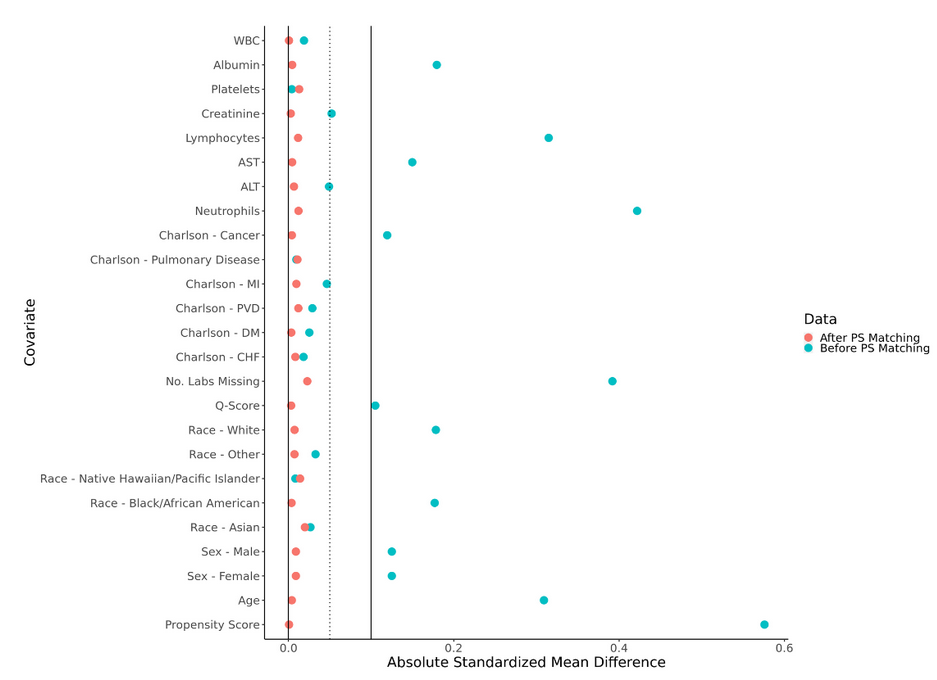

Supplement: S2 Fig — A. Assessment of PS Matching Success for Non-Remdesivir Group. Absolute standardized mean difference before and after 3:1 propensity score matching of non-dexamethasone controls to dexamethasone treated patients. All patients did not receive remdesivir. B. Assessment of PS Matching Success for Remdesivir Group. Absolute standardized mean difference before and after 1:1 propensity score matching of non-dexamethasone controls to dexamethasone treated patients. All patients received remdesivir. (ZIP) [file pone.0294892.s008.zip › S2a_Fig.tif]

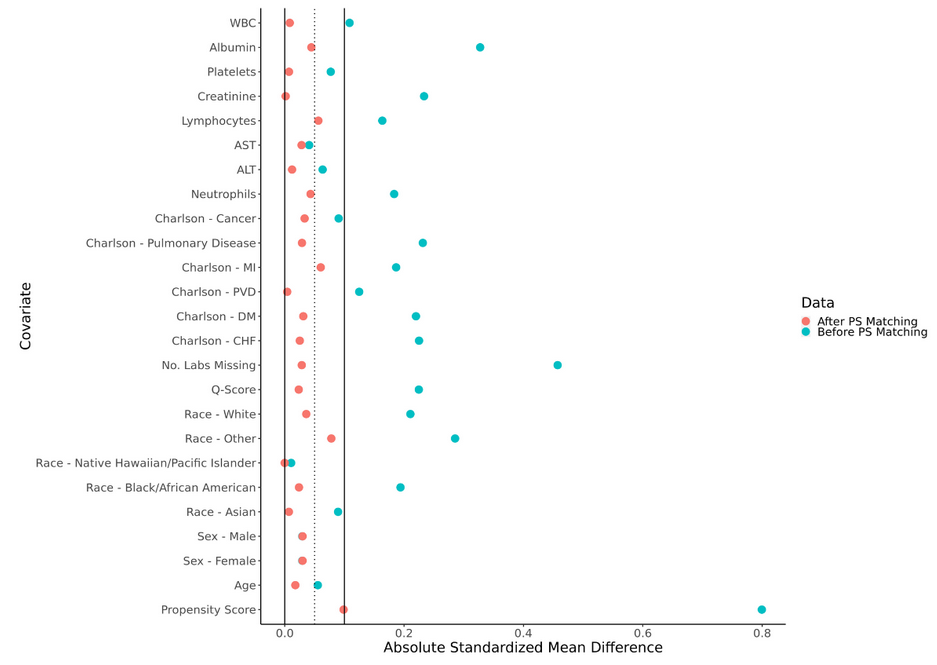

Supplement: S2 Fig — A. Assessment of PS Matching Success for Non-Remdesivir Group. Absolute standardized mean difference before and after 3:1 propensity score matching of non-dexamethasone controls to dexamethasone treated patients. All patients did not receive remdesivir. B. Assessment of PS Matching Success for Remdesivir Group. Absolute standardized mean difference before and after 1:1 propensity score matching of non-dexamethasone controls to dexamethasone treated patients. All patients received remdesivir. (ZIP) [file pone.0294892.s008.zip › S2b_Fig.tif]
